# Depletion of eIF2.GTP.Met-tRNA_i_ translation initiation complex up-regulates BRCA1 expression *in vitro* and *in vivo*

**DOI:** 10.18632/oncotarget.3125

**Published:** 2015-02-07

**Authors:** Bertal H. Aktas, Paula Bordelois, Selen Peker, Sophia Merajver, Jose A. Halperin

**Affiliations:** ^1^ Division of Hematology, Brigham and Women's Hospital, Boston, MA, USA; ^2^ Harvard Medical School, Boston, MA, USA; ^3^ Ankara University Biotechnology Institute, Ankara, Turkey; ^4^ Department of Internal Medicine, University of Michigan, Ann Arbor, MI, USA

**Keywords:** BRCA1, EPA, Fish Oil, eIF2·GTP·Met-tRNAi, uORF

## Abstract

Most sporadic breast and ovarian cancers express low levels of the breast cancer susceptibility gene, BRCA1. The BRCA1 gene produces two transcripts, mRNAa and mRNAb. mRNAb, present in breast cancer but not in normal mammary epithelial cells, contains three upstream open reading frames (uORFs) in its 5′UTR and is translationally repressed. Comparable tandem uORFs are characteristically seen in mRNAs whose translational efficiency paradoxically increases when the overall translation rate is decreased due to phosphorylation of eukaryotic translation initiation factor 2 α (eIF2α). Here we show fish oil derived eicosopanthenoic acid (EPA) that induces eIF2α phosphorylation translationally up-regulates the expression of BRCA1 in human breast cancer cells. We demonstrate further that a diet rich in EPA strongly induces expression of BRCA1 in human breast cancer xenografts.

## INTRODUCTION

BRCA1 is a major breast and ovarian cancer susceptibility gene that maintains genomic stability by regulating DNA repair/recombination and controls cell cycle progression [1]. Loss of BRCA1 function leads to carcinogenesis due to genomic instability and accelerated cell proliferation, especially when associated with either loss of function of other tumor suppressor genes, such a p53 [2] and PTEN, or overexpression of oncogenes such as erbB2 and c-myc [3–5]. Consistently, most breast and ovarian cancers express low, sometimes undetectable, levels of BRCA1 [6, 7]. Individuals carrying a germ-line mutation of the BRCA1 gene are at high risk of developing the hereditary form of breast (and ovarian) cancers, which represents approximately 3% of all breast cancer patients. In contrast, neither somatic mutations [8] nor epigenetic silencing of BRCA1 transcription by promoter methylation [6, 9–12] can fully explain the low level of BRCA1 expression frequently associated with the onset and/or progression of sporadic breast cancer, the most frequent form of the disease [13–17].

**Figure 1:**
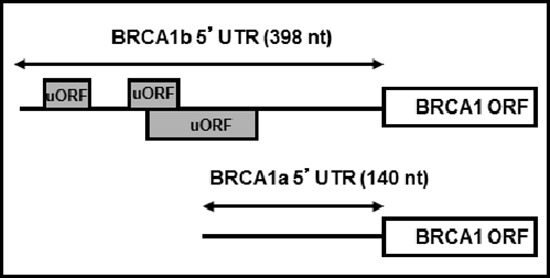
Structures of BRCA1 mRNAa and mRNAb The Figure depicts the difference in the 5′UTRs of BRCA1 mRNAa and mRNAb, highlighting the upstream ORFs in BRCA1 mRNAb.

Recent studies indicate that BRCA1 expression is regulated by micro RNAs, either directly or through their actions on other genes [18–20]. The BRCA1 expression may also be regulated by promoter switching [21]. Specifically, the BRCA1 gene is expressed under the control of two promoters, termed alpha and beta [21]. Transcription from the alpha promoter generates the BRCA1 transcript a (BRCA1 mRNAa) that contains a simple 140 nucleotide 5′UTR whereas transcription from the beta promoter generates BRCA1 transcript b (BRCA1 mRNAb) that contains an identical open reading frame (ORF) but a 398 nucleotide long 5′UTR with stable secondary structure and three upstream open reading frames (uORFs; Figure 1). The particular sequence of the BRCA1 mRNAb 5′UTR dramatically decreases its translational efficiency [22]. Evidence that normal mammary epithelial cells express BRCA1 mRNAa while cancer cells express both mRNAa and mRNAb led to the proposal that low levels of BRCA1 in the sporadic form of breast cancer could be due, at least in part, to promoter switch that favors expression of the translationally repressed BRCA1 mRNAb [22].

Two potential strategies to compensate for the reduced BRCA1 expression in breast tumors are exploiting synthetic lethal interactions between reduced BRCA1 and other cellular pathways or inducing BRCA1 expression. The latter strategy could potentially be applied to breast cancers expressing translational repressed BRCA1 mRNAb that does not carry genetic aberrations in the coding region. It is known that a subset of mRNAs with multiple uORFs is paradoxically more efficiently translated when eukaryotic translation initiation factor 2-alpha (eIF2α) is phosphorylated. Indeed, phosphorylation of eIF2α is an intriguing mechanism of translational regulation of gene-specific expression [23–25].

In the initiation phase of mRNA translation, eIF2 forms a ternary complex with GTP and initiator Methionine tRNA (eIF2·GTP·Met-tRNA_i_). This ternary complex, together with the eIF4F complex recruits the 40S ribosomal subunit to form the 43S pre-initiation complex [26]. The 43S complex scans the mRNA through the 5′UTR until it locates the initiator AUG codon where the translation-competent 80S ribosome is assembled to commence protein synthesis. Concomitantly, GTP within the ternary complex is hydrolyzed releasing the eIF2·GDP complex [27]. Initiation of a new round of translation requires recycling of eIF2·GDP to eIF2·GTP by the eIF2 guanine nucleotide exchange factor, eIF2B. Phosphorylation of eIF2α on S51 inhibits the activity of eIF2B, reduces the availability of the ternary complex and decreases the overall rate of translation initiation. Reduced availability of the ternary complex also accounts for the paradoxical increase in the translational efficiency of the subset of mRNAs with multiple uORFs [28, 29]. Characteristic examples of translational up-regulation under conditions that limit the availability of the ternary complex are the translation of yeast GCN4 and of mammalian activating transcription factor-4 (ATF-4) mRNA [24, 30, 31].

The presence and distribution of multiple uORFs in the 5′UTR of BRCA1 mRNAb (Figure 1) suggested to us that this alternative transcript, usually found in breast cancer cells, could be translationally up-regulated by agents that induce phosphorylation of eIF2α. We have demonstrated that eicosapentaenoic acid, EPA, the predominant component of n-3 unsaturated fatty acid (n-3PUFA) in marine fish oils and some chemical agents induce eIF2α phosphorylation [23, 32–36]. Furthermore, we have shown that phosphorylation of eIF2α by EPA and other compounds is directly linked to inhibition of cancer cell proliferation *in vitro* [32–34, 36], and growth of xenografted tumors *in vivo* [32–34]. Induction of eIF2α phosphorylation by EPA and chemical agents such as clotrimazole (CLT) or certain di-substituted ureas preferentially abrogates the translation of oncogenic proteins such as cyclin D1, Cyclin E and Ras and increases the expression of ATF-4 controlled genes such as CHOP [23, 32–34, 36, 37].

We report here that EPA significantly up-regulates the expression of BRCA1 in human breast cancer cells. This up-regulation occurs at the level of translation because EPA 1) does not increase either the levels of BRCA1 mRNA or the stability of the protein; 2) increases the association of BRCA1 mRNA with polysomes, and 3) up-regulates translation of reporter genes carrying the 5′UTR of BRCA1 mRNAb. These effects EPA are all dependent on phosphorylation of eIF2α because 1) they are abrogated when cells are transfected with a non-phosphorylatable mutant of eIF2α and 2) are also seen in the presence of CLT, a small molecule that phosphorylates eIF2α via a mechanism similar to EPA [38], and in the presence of di-substituted urea that induce phosphorylation of eIF2α by direct activation of the eIF2α-kinase HRI [39]. Importantly, feeding a diet rich in fish oil to mice bearing human breast cancer xenografts strongly induced expression of BRCA1 mRNA and protein in the tumors.

**Significance**: Up-regulation of BRCA1 by dietary and/or pharmacological interventions may have significant implications for the prevention and perhaps therapy of sporadic breast cancer.

## RESULTS

### EPA up-regulates the expression of BRCA1 in human breast cancer cells

**Figure 2:**
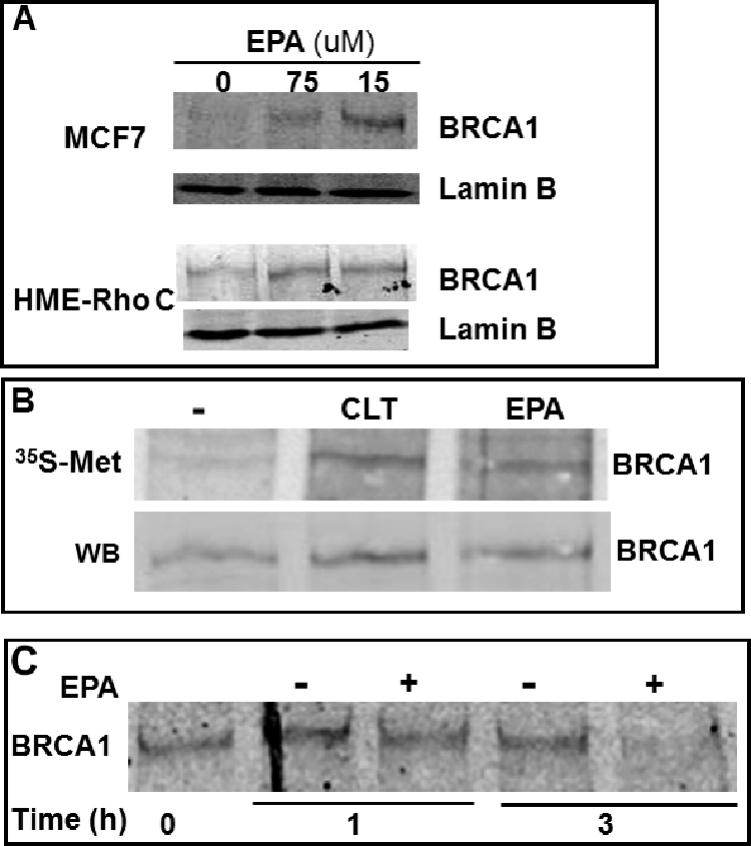
EPA translationally up-regulates BRCA1 in human breast cancer cells **(A)** MCF-7 and HME-Rho C cells were treated with indicated concentrations of EPA for 20 hours, cell lysates were separated by SDS-PAGE and immunoblotted with BRCA1 specific antibodies. **(B)** MCF-7 cells were labeled with [^35^S] Met/Cys for 15 minutes in the presence of 10 μM CLT, 150 μM EPA or DMSO. BRCA1 was immunoprecipitated and transferred to a membrane. Newly synthesized and total BRCA1 protein was visualized by Phosphoimager (top) and by Western blot (bottom), respectively. **(C)** MCF-7 cells were pulsed for 1 hr with [^35^S] Met/Cys and chased for the indicated times in the presence of DMSO or EPA. Immunoprecipitated BRCA1 was separated by SDS-PAGE and visualized by Phosphoimager.

To investigate the possibility that EPA could induce expression of BRCA1 in breast cancer cells, we used MCF-7 human breast cancer cells and HME-Rho C cells, a human breast epithelial cell line transformed by stable expression of the Rho oncogene [40], and analyzed BRCA1 expression by western blot analysis of nuclear fractions. Figure 2A shows a significant and dose-dependent increase in BRCA1 expression after exposure of MCF-7 or HME-Rho C cells to EPA. Upregulation of BRCA1 by EPA was associated with an increased incorporation of [^35^S] Met/Cys into immunoprecipitated BRCA1 (Figure 2B). This increased incorporation of [^35^S] Met/Cys was not due to delayed degradation of BRCA1 protein, as demonstrated by pulse-chase experiments that measured the half-life of BRCA1 in the presence or absence of EPA (Figure 2C). Taken together these results suggest that the EPA-induced up-regulation of BRCA1 expression is due to increased protein synthesis rather than increased stability of the protein.

### Up-regulation of BRCA1 expression occurs at the level of translation

**Figure 3:**
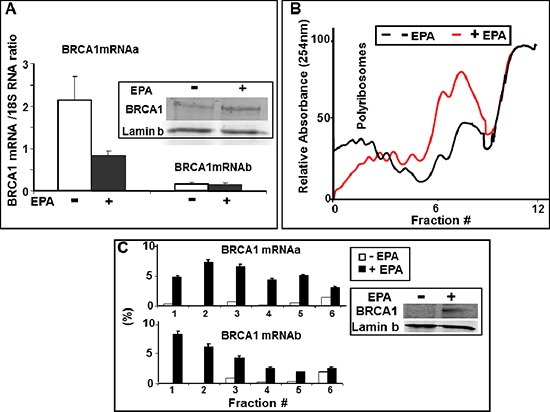
EPA translationally upregulates BRCA1 expression **(A)** Total RNA was isolated from DMSO- or 150 μM EPA-treated MCF-7 cells, and analyzed by Real Time-PCR using BRCA1 mRNAa and mRNAb specific primers. Both transcripts are normalized to the 18S RNA (arbitrary units). Mean ± SEM of four independent experiments are shown. Inset: Representative Western Blot analysis of BRCA1 in the same cells used for Real Time PCR. **(B)** MCF-7 cells were treated with DMSO (black) or EPA (150 μM, red) for 1 hour and equal OD_(260 nm)_ lysates were subjected to ultracentrifugation on 15%–50% sucrose gradients. Elution from bottom (left) to top (right) was continuously monitored at 254 nm while collecting fractions every minute. **(C)** RNA isolated from the sucrose density gradient fractions in B was reverse transcribed and amplified by Real Time PCR using BRCA1 mRNAa or mRNAb specific primers. Amplification products were quantitated with SYBR Green. Relative amounts of each transcript in individual fractions are expressed as a percentage of the total amount of the respective transcript in the lysate. Inset: Western Blot analysis of BRCA1 in the same cells used for sucrose density gradient centrifugation.

To investigate whether the observed increase in BRCA1 protein synthesis induced by EPA was due to increased accumulation of mRNA or translation, we determined in the same EPA-treated cells the protein levels of BRCA1 by Western Blot and the relative abundance of BRCA1 mRNAa and mRNAb transcripts by Real-Time PCR. Figure 3A shows that EPA induced a significant increase in the levels of BRCA1 protein without increasing the levels of either mRNAa or mRNAb transcript.

Efficiently translated mRNAs are loaded with numerous ribosomes and migrate with heavy polysomes while poorly translated mRNAs are loaded with fewer or no ribosomes and migrate with light polysomes or polysome-free fractions upon sucrose density centrifugation, an established method to assess the translation efficiency of individual mRNAs. MCF-7 cells were treated with either EPA (150 μM) or DMSO, and lysates were fractionated by sucrose density gradient centrifugation followed by analysis of BRCA1 mRNA isoforms distribution by real-time PCR. EPA treatment caused a significant shift from heavy towards lighter polysomes (Figure 3B), a confirmation that this agent inhibits translation initiation in MCF-7 cells, as we previously reported for other cell types [34, 41]. Furthermore, real-time PCR analysis of BRCA1 mRNAs in the individual fractions from the polysome profiles in Figure 3B showed that treatment with EPA increased the association of both BRCA1 mRNAa and mRNAb with polysomes, as demonstrated by the increase in their relative abundance in the heavier fractions of the gradient (fractions 1 to 4). This shift was particularly prominent in heavy polysomal fractions (fractions 1 and 2) and more pronounced for the transcript b than the transcript a (Figure 3C). Indeed, the cumulative content of BRCA1 mRNAb in fractions 1and 2 increased from 0.2 to 15% whereas the content of BRCA1 mRNAa transcript increased from 0.5 to 11.5%, representing a 75- and 23-fold increase, respectively. These results are consistent with the increased expression of the BRCA1 protein, and confirm the translational up-regulation of BRCA1 mRNA. The polysome profiles in Figure 3B also showed a very low abundance of both BRCA1 mRNAa and mRNAb in the polysomes fractions of untreated cells, suggesting that in MCF-7 cells both BRCA1 transcripts are translated with low efficiency.

### Translational up-regulation of BRCA1 *in vitro* is mediated by phosphorylation of eIF2α and depletion of the ternary complex

**Figure 4:**
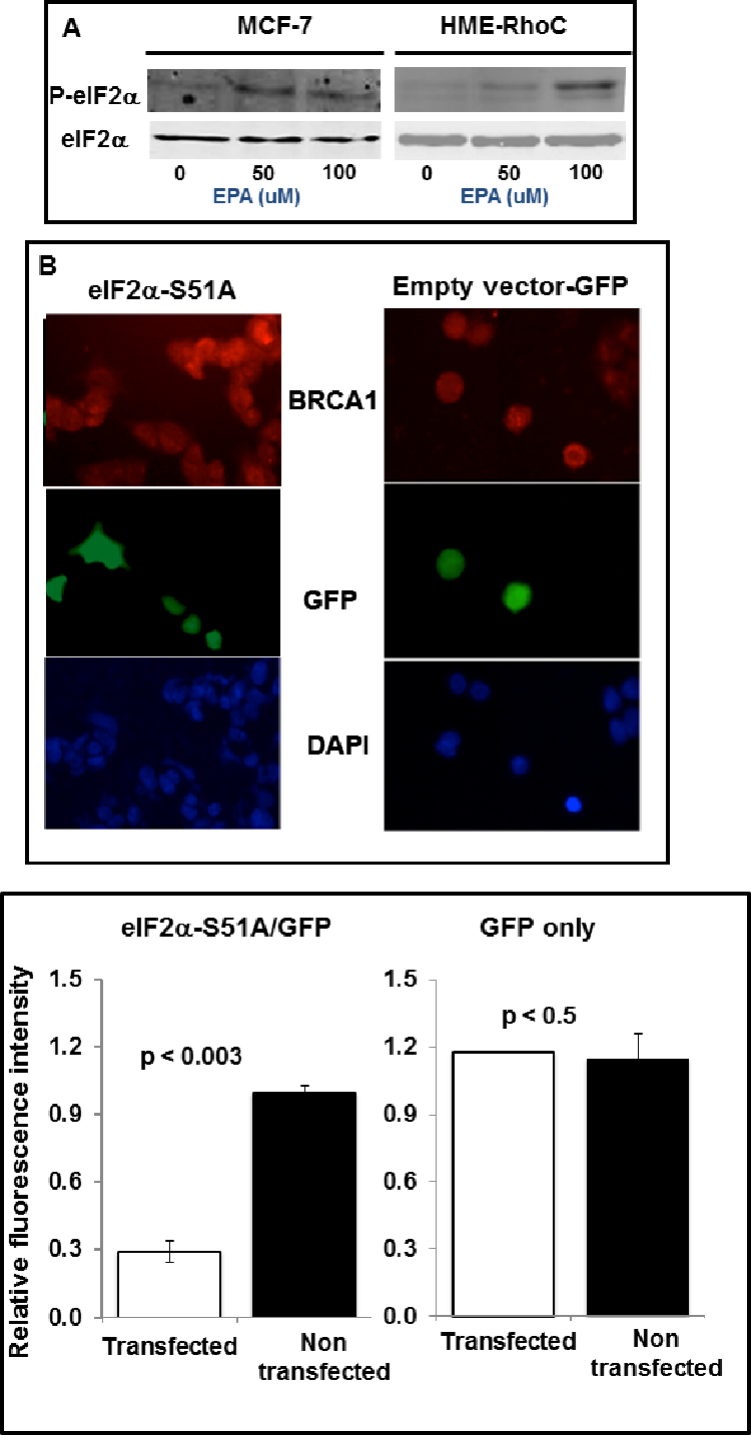
Up-regulation of BRCA1 in breast cancer cells depends on phosphorylation of eIF2α **(A)** Western blot analysis of P-eIF2α and total eIF2α in MCF-7 and HME-RhoC cells treated with the indicated concentrations of EPA. **(B)** MCF-7 cells transfected with GFP-eIF2α51A (left panels) or with GFP-empty vector (right panels), were treated with EPA, fixed with 4% formaldehyde, permeabilized with 0.1 Triton X-100, and stained with anti-BRCA1 antibody (top panels) or DAPI (bottom panels). The middle panels show GFP-eIF2αS51A or GFP-empty vector transfected cells. **(C)** Quantification of data from experiment shown in B.

We have previously demonstrated that EPA induces phosphorylation of eIF2α and thereby depletion of the ternary complex and inhibition of translation-initiation [34, 35]. Figure 4A shows increased phosphorylation of eIF2α one hour after treatment of MCF-7 or HME-Rho C cells with EPA. This indicates that up-regulation of BRCA1 expression by EPA, as shown in Figure 2, is associated with eIF2α phosphorylation.

To formally demonstrate the cause-effect relationship between phosphorylation of eIF2α and increased expression of BRCA1, we transiently transfected MCF-7 cells with a bidirectional expression vector that codes for the reporter protein GFP on one arm and on the other for the non-phosphorylatable eIF2αS51A mutant [38]. We assessed the expression level of BRCA1 by immunocytochemical analysis of EPA-treated cells (Figure 4B). The microscopic fields contained cells transfected with the non-phosphorylatable GFP-eIF2α S51A mutant (left) or with GFP-empty vector (right), identified in the middle panels of Figure 4B by the green fluorescence emitted by GFP, as well as non-transfected cells. Both transfected and non-transfected cells can be seen by DAPI staining in the bottom panels of Figure 4. The pictures show significantly lower expression of BRCA1 in the EPA treated GFP-eIF2αS51A expressing cells (green cells in the Figure 4B left panels) than in adjacent cells in the same panel that express only endogenous eIF2α (i.e. cells that do not express GFP in the same panels) or cells transfected with only GFP encoding-vector independently of GFP expression (Figure 4B, right panels). These results, shown quantitatively in Figure 4C, demonstrate that EPA-induced up-regulation of BRCA1 is mediated by phosphorylation of eIF2α.

### Structural features of its 5′UTR may contribute to the translational up-regulation of BRCA1 mRNAb

**Figure 5:**
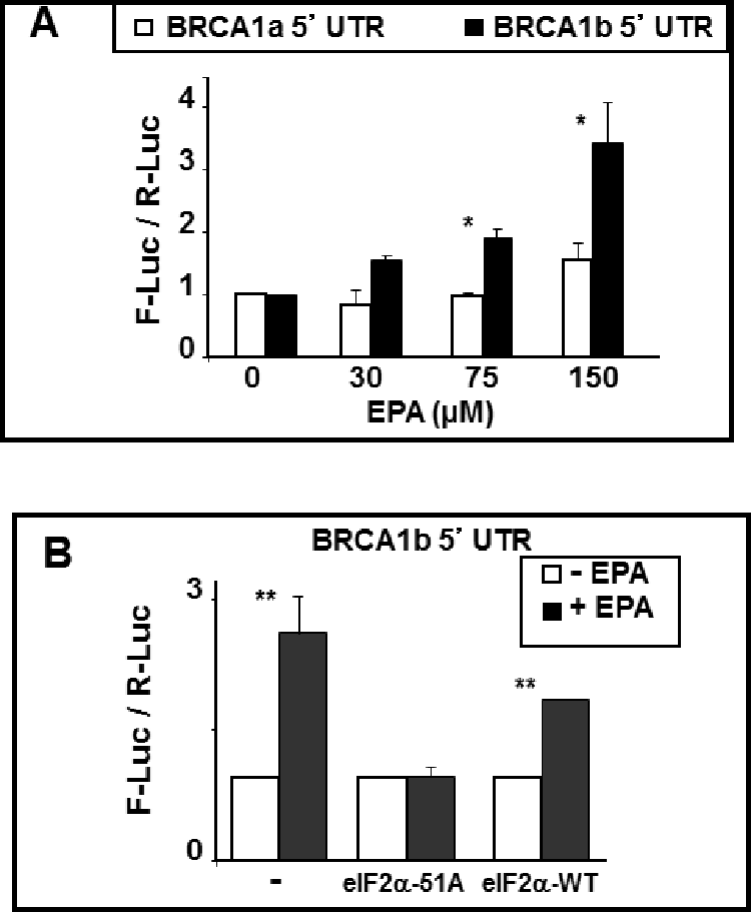
BRCA1 mRNAb 5′UTR confers EPA-induced translational up-regulation to reporter genes **(A)** BRCA1 mRNAa or mRNAb 5′UTRs (including the first three codons of BRCA1 ORF) were fused in-frame to the ORF of F-Luc in the bidirectional pHGB^F-Luc / R-Luc^ plasmid. The same 90 nucleotide plasmid-derived 5′UTR preceeds the renilla ORF and the 5′UTR of either mRNAa or mRNAb in the final plasmid. MCF-7 Tet-off cells were transiently transfected with either of the plasmids, treated with DMSO or EPA overnight and reporter activities were determined by dual luciferase assay. Bars indicate Mean ± SEM of F-Luc / R-Luc ratios in treated cells normalized to DMSO controls. *p < 0.05. **(B)** MCF-7 cells were co-transfected with the reporter plasmids described in A and either eIF2α-S51A or eIF2α-WT expression plasmids. Cells were treated with DMSO or EPA and processed as in A. Bars indicate Mean ± SEM of F-Luc / R-Luc ratios in treated cells normalized to DMSO controls. **p < 0.01.

To explore the role of the mRNAa and mRNAb 5′UTRs in the EPA-mediated translational up-regulation of BRCA1, we constructed two plasmids containing a bi-directional tetracycline-inducible promoter flanked on one side by a minimal 90 bp 5′UTR sequence followed by the renilla luciferease (R-Luc) ORF and on the other side by the same 90 bp plus either the 141 bp or the 398 bp 5′UTRs of BRCA1 mRNAa and mRNAb, followed by the ORF of firefly luciferase (F-Luc). MCF-7 Tet-off cells were transiently transfected with these plasmids, challenged with EPA 48 hours after transfection for additional 16 hours, and the reporter gene activities determined by the dual luciferase assay. To account for different transfection efficiency between wells, we expressed the results as F-Luc/R-Luc ratio in all EPA-treated wells normalized to same ratios in the DMSO-treated control wells in each plate. Ratios higher than 1 are indicative of increased translation of the respective F-Luc mRNA. The results showed that EPA significantly increased the expression of F-Luc when its ORF was preceded by the 5′UTR of BRCA1 mRNAb whereas it was not significantly affected when preceded by the 5′UTR of mRNAa (Figure 5A). This sensitivity to EPA-mediated up-regulation conferred onto the reporter gene by the 5′UTR of BRCA1 mRNAb was abrogated by co-transfection of the cells with the non-phosphorylatable eIF2αS51A mutant. In contrast, EPA increased the expression of the reporter gene in cells co-transfected with the wild type eIF2α (Figure 5B). Thus, the 5′UTR of the BRCA1 mRNAb seems to contain structural features that make this mRNA susceptible to translational up-regulation under conditions that induce phosphorylation of eIF2α and reduce the amount of the ternary complex.

**Figure 6:**
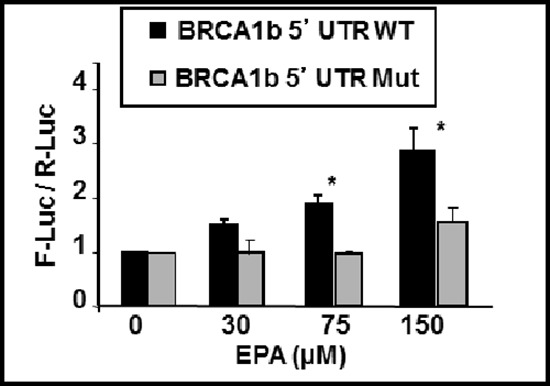
EPA-induced translational up-regulation of reporter genes fused to BRCA1 mRNAb 5′UTR is dependent on the presence of tandem uORFs The plasmid encoding for F-luc fused to the 5′UTR of BRCA1 mRNAb with (BRCA1b 5′UTR Mut) and without (BRCA1b 5′UTR WT) mutations that replaced the three AUG codons for non-initiator AAG codons were transfected to MCF-7 cells. Cells were treated with EPA or DMSO and reporter activity was measured by dual luciferase assay. Bars indicate Mean ± SEM of F-Luc / R-Luc ratios in treated cells normalized to DMSO controls. *p < 0.01.

To confirm that these effects were due to the presence of uORF in the 5′UTR of BRCA1 mRNAb, we utilized a mutant of the BRCA1b 5′UTR where all three uAUGs in the uORFs (shown in Figure 1) were mutated to A**A**G (Mut-5′UTRb, kindly provided by Dr. Sobczak). Since AAG codon does not initiate translation, it removes the tandem uORFs in the 5′UTRb sequence. Figure 6 shows that fusion of the mutant BRCA1 mRNAb 5′UTR to F-luc abrogated EPA-induced up-regulation of this reporter (Figure 6). Taken together, these results provide strong support to our contention that structural features in the 5′UTR of BRCA1 mRNAb may contribute to its translational up-regulation by EPA through reduced availability of the ternary complex.

### Fish oil rich in EPA induce up-regulation of BRCA1 *in vivo*

**Figure 7:**
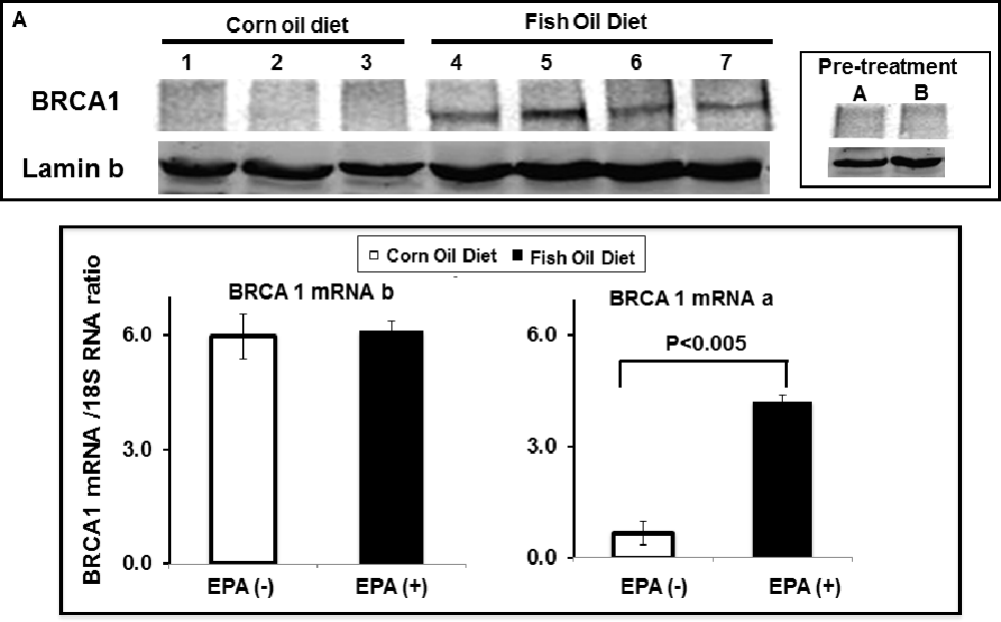
EPA up-regulates BRCA1 *in vivo* MCF-7 human breast cancer cells were orthotopically xenographted in nude mice. Mice were randomly distributed to two groups, fed with either corn- (1–3) or fish-oil (4–7) supplemented diet for 3 weeks, tumors were excised and analyzed for BRCA1 protein expression by western blot **(A)** and for BRCA1 mRNA expression by Real Time PCR **(B)** using BRCA1 mRNAa and mRNAb specific primers. Levels of both transcripts are normalized to 18S RNA.

The potential effect of EPA on the BRCA1 expression *in vivo* was tested in an orthotopic xenograft model of MCF-7 human breast cancer cells. Tumors were formed by implanting the cancer cells into the mammary fat pad of female athymic nude mice. After allowing the tumors to grow to a measurable size (400 mm^3^), the animals were randomized into 2 groups and fed an equicaloric fish-oil or corn-oil rich diet. These diets were identical in all components except for their fatty acid composition: The fish oil diet contained 19% manhedan (a fish oil rich in EPA) and 1% corn oil), while the corn oil diet contained 20% corn oil (the small amount of corn oil in the “fish oil” diet provides the daily requirement of essential fatty acids not contained in the Menhaden oil. After three weeks, animals were sacrificed, the tumors were excised and analyzed for levels of BRCA1 protein by Western blot and BRCA1 mRNA by real-time PCR (Figure 7).

The results showed that there was a much higher level of BRCA1 protein in tumors excised from mice fed with the fish-oil diet than in tumors excised from mice fed with the corn-oil diet (Figure 7A). Sentinel mice sacrificed before starting the diets showed undetectable levels of BRCA1. Real-time PCR analysis of the tumors excised after treatment with corn or fish oil diet revealed no differences in levels of mRNAb while mRNAa was mildly increased in the mice receiving fish-oil diet (Figure 7B). These results suggest that EPA induces up-regulation of BRCA1 *in vivo* as it does *in vitro* and that increased expression of BRCA1 mRNAa may contribute to the expression of BRCA1 protein *in vivo*.

### Structurally and mechanistically divergent chemical agents that cause eIF2α phosphorylation induce BRCA1 expression

To further confirm that EPA upregulates BRCA1 expression by inducing eIF2α phosphorylation we repeated key experiments described above with clotrimazole (CLT), an agent that similar to EPA induces eIF2α phosphorylation by partially depleting endoplasmic reticulum Ca^++^ stores [34, 38]. As expected, CLT induced expression of BRCA1 in both MCF-7 and HME-Rho C breast cancer cells (Supplemental Figure 1). CLT also increased expression of a reporter gene fused to the 5′UTR of BRCA1 mRNAb but not BRCA1 mRNAa (Supplemental Figure 2A) in an eIF2α phosphorylation dependent manner (Supplemental Figure 2B). Consistently, CLT induced expression of a reporter gene fused to the wild type 5′UTR of BRCA1 mRNAb but not mutant 5′UTR of BRCA1b mRNA in which the AUG codon of all three uORF were replaced with AAG thereby eliminating all three uORFs (Supplemental Figure 3). These data provide additional support to our contention that at least *in vitro*, EPA induces BRCA1 expression by causing phosphorylation of eIF2α thereby reducing the amount of the eIF2·GTP·Met-tRNA_i_ ternary complex.

To rule out an unlikely possibility that partial depletion of endoplasmic reticulum Ca^++^ stores may be responsible for increasing BRCA1 expression independently of or in addition to eIF2α phosphorylation, we tested another small molecular agent, Compound 2 (C#2) [35], that also induces eIF2α phosphorylation. We chose C#2 because this class of *N,N’*-diarlyureas cause eIF2α phosphorylation by directly activating heme regulated inhibitor [33, 35], one of the four eIF2α kinases. As shown in Supplemental Figure 4, C#2 also upregulated BRCA1 expression, further demonstrating that phosphorylation of eIF2α and reducing the amount of the eIF2·GTP·Met-tRNA_i_ ternary complex induces expression of BRCA1 in breast cancer cells.

## DISCUSSION

The work reported here demonstrates that EPA translationally up-regulates the expression of BRCA1 in human breast cancer cells *in vitro* and in human breast cancer xenografts.

The presence of multiple uORFs, together with excessive secondary structure in the 5′UTR, is one of the key structural factors that decrease the translational efficiency of mRNAs [42–44]. However, there is a small subpopulation of mRNAs in which the configuration of uORFs in the 5′UTR has evolved in a manner that restricts translation when the eIF2×GTP×met-RNA ternary complex is abundant but paradoxically favors their translation under conditions - such as increased phosphorylation of eIF2α - that reduce the amount of the ternary complex [28–30, 45–48]. The work reported here indicates that the structural configuration of uORFs present in the 5′UTR of BRCA1 mRNAb may render this transcript susceptible to translational up-regulation under conditions that limit the abundance of the ternary complex.

Extensive work from our laboratory has demonstrated that EPA, CLT, and certain *di*-substituted urea analogs [33, 35, 36] induce phosphorylation of eIF2α and thereby reduce the amount of the ternary complex. We have further shown that reducing the amount of the ternary complex downregulates expression of oncogenic proteins such as cyclin D1 and up-regulates expression of ATF-4 and genes under its transcriptional control [32, 34, 35]. For this reason, several key experiments originally conducted with EPA were repeated with CLT and one with C#2; comparable results with structurally different agents that reportedly induce phosphorylation of eIF2αvia different mechanisms, support the conclusion that phosphorylation of eIF2αis plays an important role in the observed effects of EPA on BRACA1 expression at least *in vitro*.

The results of the experiment depicted in Figures 2 and 3 demonstrate that the significant up-regulation of BRCA1 protein induced by exposure to EPA is due to increased synthesis rather than stability of BRCA1 protein because 1) it is associated with an increased incorporation of [^35^S] Met/Cys which requires de novo protein synthesis, 2) it is not associated with an extended half-life of BRCA1 protein, as demonstrated by pulse-chase experiments and 3) it is not associated with a parallel increase in BRCA1 mRNA levels measured by real time PCR (Figure 3A). The translational up-regulation of BRCA1 occurs at the level of initiation, as demonstrated by the increased association of BRCA1 mRNA with polysomes separated by sucrose density gradient (Figure 3C). Treatment with EPA induces a significant shift of BRCA1 mRNA towards fractions containing heavy polysomes, an indication of enhanced translation initiation. This effect is more pronounced for BRCA1 mRNAb but is evident for the BRCA1 mRNAa as well.

The increased translation initiation of the BRCA1 mRNA *in vitro* is dependent of eIF2α phosphorylation; in the case of mRNAb it also depends on the presence of multiple uORFs in its 5′UTR. These conclusions are based on the results of experiments showing that a) expression of the constitutively active and non-phosphorylatable eIF2αS51A mutant (Figure 4B) abrogates up-regulation of both the BRCA1 protein and the luciferase reporter fused to the 5′UTR of BRCA1 mRNAb (Figure 5B). Comparable results were obtained when phosphorylation of eIF2α was induced with compounds that cause either partial depletion of endoplasmic reticulum Ca^++^ stores, such as CLT or EPA, or directly activate heme regulated inhibitor kinase, such as C#2. Compounds that induce phosphorylation of eIF2α (Figure 4A), inhibit translation initiation because they reduce the amount of the ternary complex, as demonstrated by the increased expression of ATF-4 dependent genes such as CHOP [23, 32, 34, 35]. All these downstream effects of eIF2α phosphorylation are abolished by the expression of eIF2α-S51A [49] as we now show for EPA-induced up-regulation of BRCA1 (Figure 4B). In the case of BRCA1 mRNAb transcript this susceptibility to translational up-regulation by eIF2α phosphorylation is at least in part dependent on the structure of its 5′UTR, which contains three uORFs, as demonstrated by the reporter-gene assay depicted in Figure 5. The F-luc reporter gene becomes susceptible to phosphorylated eIF2α dependent up-regulation when preceded by the 5′UTR of BRCA1 mRNAb but not when preceded by the 5′UTR of BRCA1 mRNAa or by a 5′UTR mRNAb mutant in which the upstream initiator AUG codons were changed to non-initiator AAG codons.

In contrast to BRCA1 mRNAb, the increased association of the mRNAa transcript with polyribosomes (Figure 3C) does not seem to be mediated by structural features of its 5′UTR. Fusion of mRNAa 5′UTR to reporter gene does not render the expression of this reporter sensitive to EPA. The molecular basis of increased recruitment of the BRCA1 mRNAa to ribosomes in breast cancer cells treated by EPA is not known at the present time.

One important finding of our studies is that an EPA rich fish-oil containing diet but not an equal-caloric corn oil containing diet induces up-regulation of BRCA1 protein in MCF-7 xenograft tumors. This result may potentially explain, at the molecular level, previously reported observations that fish-oil enriched diets inhibit growth of human breast xenografts [50, 51]. It has been shown that the promoter of BRCA1 mRNAa contains a PPARγ binding site and that the PPARγ agonist roziglitazone induces transcription of BRCA1 mRNAa from the alpha promoter [52]. Since EPA and perhaps its metabolites are also PPARγ agonists [53, 54] the increased expression of BRCA1 mRNAa in the breast cancer xenografts of mice fed the fish-oil supplemented diet could be explained, at least in part, by increased transcription. Given these complexities, we cannot determine at the present time the relative contribution of translation and transcription to the increased expression of BRCA1 observed in xenografts derived from EPA-treated mice. Further studies are needed to understand how an EPA rich fish-oil supplemented diet increases expression of BRCA1 mRNA and protein.

In summary, the results of this work may have implications for breast cancer prevention and perhaps treatment. A body of experimental and clinical evidence indicates that BRCA1 levels influence the susceptibility of women to develop breast cancer as well as the aggressiveness of the tumors. Women carrying a germ-line mutation of BRCA1 are at much higher risk of developing an aggressive form of breast cancer when they are still young. In the sporadic form of breast cancer levels of BRCA1 in the excised tumors seems to correlate inversely with the aggressiveness and metastatic potential of the tumors [13, 14]. It is conceivable that in the progressive transformation from normal to malignant cells in sporadic breast cancers, the relative abundance of the inefficiently translated BRCA1 mRNAb transcript may play an role in the reduced expression of BRCA1 protein. In turn, low abundance of BRCA1 would increase the genomic instability, increase the proliferation and decrease the differentiation of the tumor cells, all contributing factors to the malignant phenotype of a tumor. In this context, an intervention that increases the expression of BRCA1, as documented in this paper for mice bearing human breast tumors fed with a diet rich in EPA, has the potential of delaying and/or preventing the transformation and perhaps suppressing the aggressiveness of human breast cancers. A meta-analysis of published studies on the effect of omega-3 PUFAs on the risk of human cancers found that 7 out of the 11 breast cancer studies analyzed did not show a significant association [55]. The results reported here indicate that both phosphorylation of eIF2α and levels of BRCA1 may provide needed biomarkers for future clinical studies assessing the potential effect of n-3 PUFAs on breast cancer risk and perhaps identifying individuals that may benefit from dietary intervention.

This work also opens the possibility of utilizing non-dietary agents that cause phosphorylation of eIF2α for prevention and/or treatment of breast cancers that express high level of BRCA1 mRNA but not protein. Several novel chemical agents that activate eIF2α kinases have recently been identified [33, 36, 37]. The availability of the three dimensional structure of eIF2α kinases PKR and PERK [56–58] could further sped up the development of these agents.

## METHODS

### Ethics statement

All animal studies in this report are carried out per Harvard Medical School Standing Committee on Animals, IACCUC that oversees studies involving animals per approved protocol #03151.

### Cell culture

MCF-7 Tet-off cells (Clontech) were maintained in RPMI medium (Gibco) supplemented with 10% Fetal Bovine Serum (FBS) and antibiotics. HME-Rho C cells were maintained as described [40]. For treatment, FBS was reduced to 5%. All cells were cultured at 37°C in 5% CO_2_.

### Immunoblotting

Nuclear extracts were prepared with Nuclear Extraction Kit (Active Motif) and protein concentration were measured by BCA Protein Assay (Pierce). Extracts were separated by 6% SDS-PAGE, transferred to nitrocellulose membranes (Bio Rad Laboratories) and immunoblotted with: mouse monoclonal anti-BRCA1 antibody (D-9, Santa Cruz), goat polyclonal anti-Lamin B antibodies (M-20, Santa Cruz); rabbit polyclonal (Stressgen) or rabbit monoclonal anti-P-eIF2α antibodies (Epitomics), and mouse monoclonal anti-eIF2α antibody (Biosource). Secondary antibodies were IRDye 800 Conjugated anti-mouse, IRDye 800 Conjugated anti-goat (Rockland), and Alexa-Fluor 680 anti-rabbit (Molecular Probes) antibodies. An Odyssey Infrared Imaging System (Li-Cor Biosciences) was used to develop and quantify immunoblots.

### RNA isolation and quantification

The RNeasy Mini Kit (Qiagen) was used for RNA isolation following manufacturer's instructions. DNAse digestion was performed in-column with Rnase-Free Dnase Set Kit (Qiagen) and off-column with Deoxyribonuclease I (Invitrogen).

### cDNA synthesis and real time PCR

ThermoScript RT-PCR System (Invitrogen) was used for cDNA synthesis with a mixture of Oligo (dt)_20_ primers and Random Hexamers.

Real Time PCR was conducted in an iCycler instrument (Bio-Rad Laboratories) using SYBR Green PCR kit (Qiagen) for rapid analysis of targets.

Primers for both BRCA1 transcripts were designed specifically to span from exon 1a or 1b to exon 2. BRCA1-a primers: F: 5′-CAGGCTGTGGGGTTTCTCA-3′, R: 5′-TCAACGCGAAGAGCAGATAAAT-3′, BRCA1b primers: F: 5′-TGGGCGACAGAGCGAGACT-3′, R: 5′-CAACGCGAAGAGCAGATAAATCC-3′; 18S RNA primers: F: 5′-CGGCGACGACCCATTCGAAC-3′ and R:5′-GAATCGAACCCTGATTCCCCGTC-3′,

No-RT processed aliquots of the samples were used to control for potential genomic DNA contamination.

Cycling conditions were: 95°C for 15 minutes followed by 45 cycles of 94°C 30 seconds, 60°C 30 seconds and 72°C 30 seconds. All samples were analyzed in quadruplicates, and experiments were replicated four times.

### Plasmids and constructs

The 5′UTRs of BRCA1 mRNA a and b were amplified from a human testis cDNA library (Clontech) and cloned into the bi-directional pHGB^fluc/rluc^ plasmid [59] between the MluI and XhoI sites of the MCSI 5′ upstream to the Firefly ORF. Primers used for amplification of BRCA1 5′UTRa were: forward 5′-CAAACGCGTAAAACTGCGACTG CGCGGC-3′ and reverse 5′-TCCCTCGAGGCGAAGAGCAGATAAATC CAT-3′. Primers for amplification of BRCA1 5′UTRb were: forward 5′-TTAACGCGTGGGCAGTTTGTAGG TCG-3′ and reverse 5′-TCCCTCGAGGCGAAGAGCAGA TAAATCCAT-3′.

Mutant BRCA1b (Mut-BRCA1) 5′UTRb was obtained by re-amplification of a PCR product kindly provided by K. Sobczak and cloned into pHGB^fluc/rluc^, as described above.

GFP-S51A and GFP-eIF2αWT constructs: GFP and eIF2α-S51A and eIF2αWT are described in [39].

### Dual luciferase assay

MCF7-tet off cells seeded in 24 well plates (20.000/well) were transfected with 20 ng of plasmid DNA/well with Effectene kit (Qiagen), following manufacturer instructions. Transfection complexes were removed from the plates 12 hours after transfection, and cells were treated with DMSO, EPA or CLT at doses indicated in the figures. Luciferase activity in cell lysates was detected with the Dual-Glo Luciferase kit (Promega).

Transfection efficiency was controlled by normalizing the Firefly/Renilla ratio in each experimental well to the ratio in the DMSO treated wells in the same plate. All experiments were conducted in triplicates.

### Protein synthesis ([^35^S]Met/Cys incorporation)

Exponentially growing cells were washed with and pre-incubated in Met-Cys-free RPMI (MP Biosmedicals) for 45 minutes followed by a 15 minutes treatment with DMSO, EPA or CLT in the same medium containing [^35^S]Met/Cys (200 uCi/mL; Easy Tag Express). Cells were washed with ice-cold PBS, harvested and lysed with RIPA buffer. Total protein was quantified by the BCA method and aliquots containing equal amounts of protein were immunoprecipitated with goat anti-BRCA1 antibody (Stressgen) and separated by SDS-PAGE followed by autoradiography and Western blot analysis [37].

### Polysome profiles

Exponentially growing MCF-7 cells were treated with DMSO or EPA for 1 h before addition of cycloheximide (100 uM) for 5 min. Cells were washed with cycloheximide containing ice-cold PBS, harvested and lysed. Equal OD_(260 nm)_ aliquots were subjected to sucrose density gradient (15–50%) centrifugation, the gradient was eluted under continuous monitoring at 254 nm collecting one minute fractions [38]. RNA in each fraction was isolated and processed for quantification of BRCA1 transcripts by Real time PCR.

### Pulse-chase experiments

Exponentially growing MCF-7 cells were cultured in methionine and cysteine free media for one hour, then pulsed with 250 uCi/mL of [^35^S]Met/Cys for 1 h followed by washing of raio-labeled media and chasing in complete media supplemented with 70X molar excess cold methionine and cysteine in the presence or absence of EPA. Aliquots containing equal amount of protein were immunoprecipitated, separated by SDS-PAGE and ^35^S labeled proteins were visualized and quantified with a Phosphoimager.

### Immunostaining

Cells were seeded in 4 wells Lab-Tek chamber slides (Nunc) and allowed to recover for 16–18 hours before exposure to EPA for 1 hour. Cells were fixed with 4% paraformaldehyde for 20 min, permeabilized with 0.2% Triton-X100 for 15 min, and blocked with 3% BSA/1% normal goat serum for 1 hour. After fixation, cells were exposed to the Ab1 anti-BRCA1 antibody (Calbiochem) overnight (1:140 dilution) in a humidified chamber. Secondary antibody was goat anti-mouse Alexa Fluor 546 (Molecular Probes) used at 1:400 dilution. Immunofluorescence was detected and recorded with a Nikon TE 2000-E microscope.

### Orthotopic xenograft model of breast cancer

MCF-7 cells were trypsinized, washed, and resuspended in Hank's buffered saline solution (HBSS)/Matrigel (50:50 v/v) at a density of 1 × 10^6^ cells/100 μl. Female athymic nude mice (8–10 weeks old) were anesthetized using 10 mg/ml ketamine, 1 mg/ml xylazine, and 0.01 mg/ml glycopyrrolate, before exposing the mammary fat pad through an incision below the thoracic left mammary gland. The cell suspension was injected into the mammary fat pad and the wound closed with a single wound clip. At the time of tumor injection, mice were also implanted subcutaneously on the dorsal surface with a 17-β-estradial pellet (1.7 mg/pellet; 60 day release) (Innovative Research of America). At 4 weeks post-tumor grafting, mice with established tumors of approximately 0.4 cm^3^ were randomly assigned to two experimental protocols and fed with equicaloric diets that differ only for the source of fatty acids, which were supplied from either corn or fish (Menhaden) oil. Four weeks later, animals were sacrificed, tumors were surgically removed and stored in RNAlater buffer.

## SUPPLEMENTAL FIGURES


