# Supplementary material for: Depletion of eIF2.GTP.Met-tRNAi translation initiation complex up-regulates BRCA1 expression in vitro and in vivo
Source: Oncotarget. 2015 Feb 7;6(9):6902–14. doi: 10.18632/oncotarget.3125 (PMC4466658; doi:10.18632/oncotarget.3125)
Supplement: Supplementary file 1 [file oncotarget-06-6902-s001.pdf]

SUPPLEMENTAL FIGURES

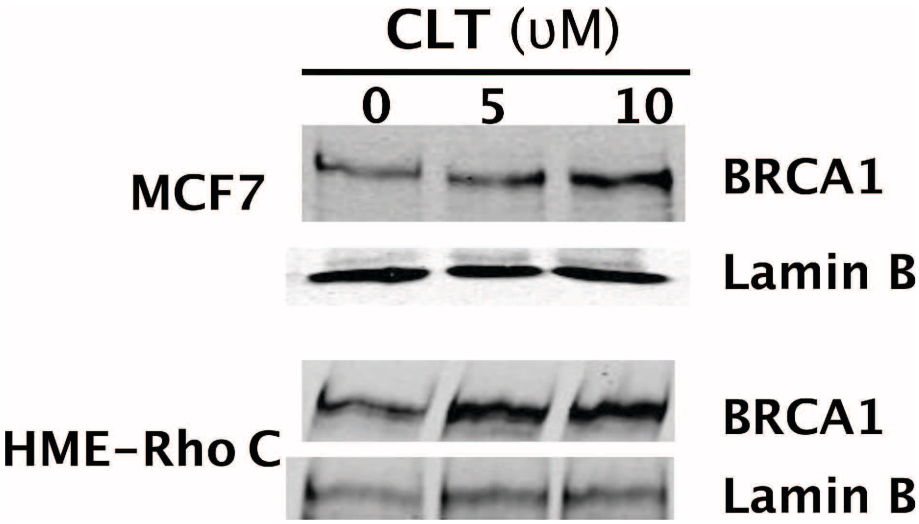

Supplemental Figure 1: CLT up-regulate BRCA1 expression in human breast cancer cells. MCF-7 and HME-Rho C cells were treated with indicated concentrations of CLT for 20 hours, and cell lysates were immunoblotted with BRCA1 specific antibodies.

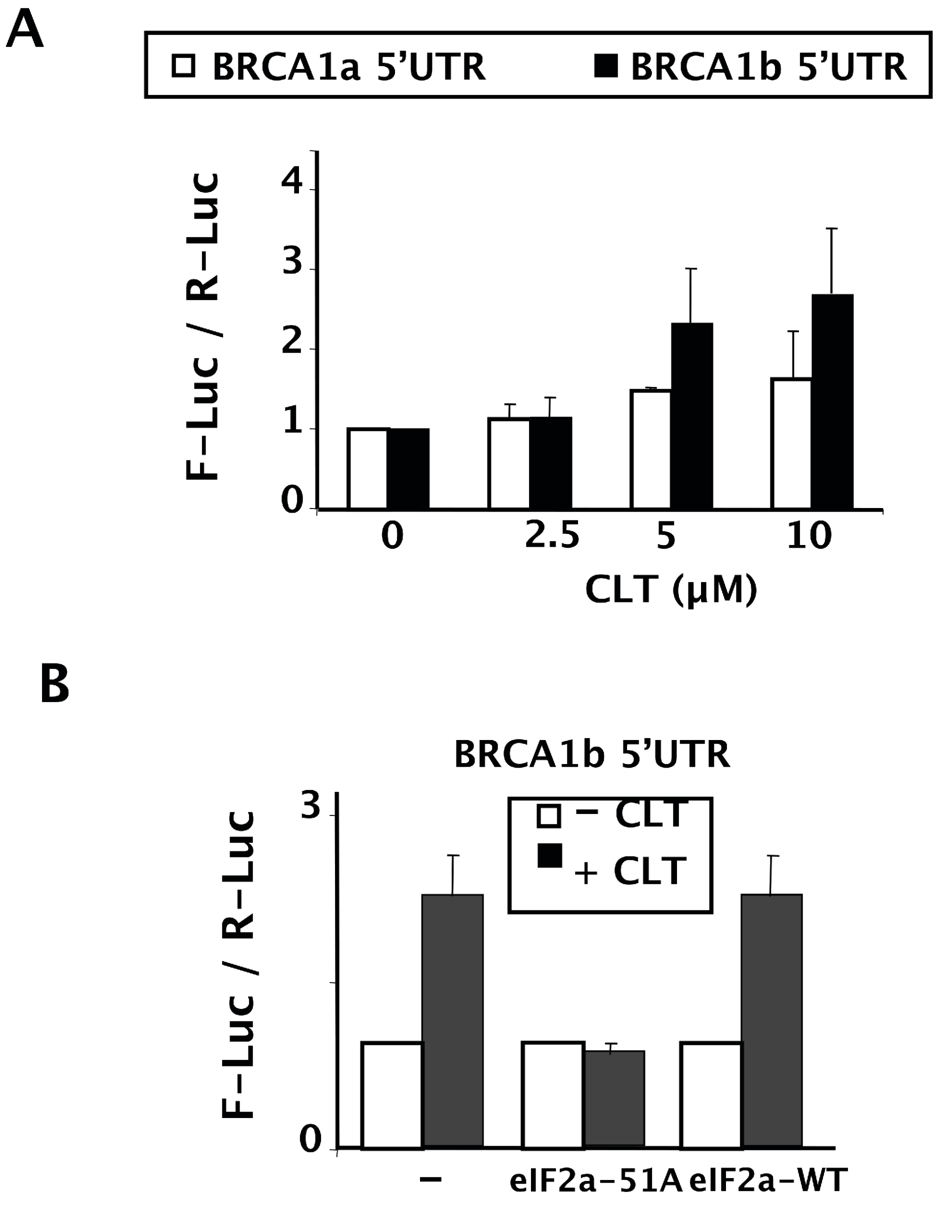

Supplemental Figure 2: BRCA1 mRNAb 5'UTR confers EPA-induced translational up-regulation to reporter genes. (A) BRCA1 mRNAa or mRNAb 5'UTRs (including the first three codons of BRCA1 ORF) were fused in-frame to the ORF of F-Luc in the bidirectional pHGB$^{F\text{-}Luc\,/\,R\text{-}Luc}$ plasmid as described in Figure 5. MCF-7 Tet-off cells were transiently transfected with either of the plasmids, treated with DMSO or CLT overnight and reporter activities were determined by dual luciferase assay. Bars indicate Mean ± SEM of F-Luc / R-Luc ratios in treated cells normalized to DMSO controls. **(B)** MCF-7 Tet-off cells were co-transfected with the reporter plasmids described in A and either eIF2α-S51A or eIF2α-WT expression plasmids. Cells were treated with DMSO or CLT and processed as in A. Bars indicate Mean ± SEM of F-Luc / R-Luc ratios in treated cells normalized to DMSO controls.

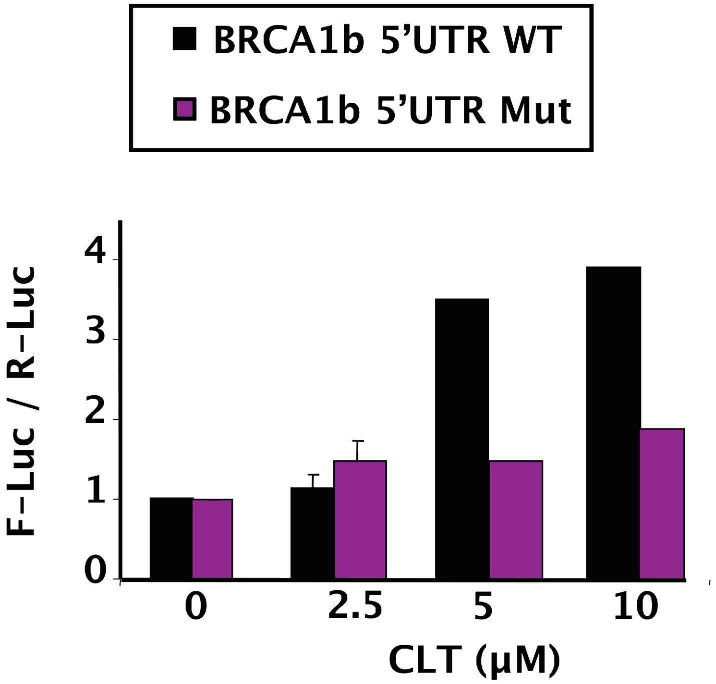

Supplemental Figure 3: CLT-induced translational up-regulation of reporter genes fused to BRCA1 mRNAb 5'UTR is dependent of the presence of tandem uORFs. The plasmid encoding for F-luc fused to the 5'UTR of BRCA1 mRNAb with (BRCA1b 5'UTR Mut) and without (BRCA1b 5'UTR WT) mutations that replaced the three upstream AUG codons for non-initiator AAG codons were transfected to MCF-7 cells. Cells were treated with CLT or DMSO and reporter activity was measured by dual luciferase assay. Bars indicate Mean ± SEM of F-Luc / R-Luc ratios in treated cells normalized to DMSO controls.

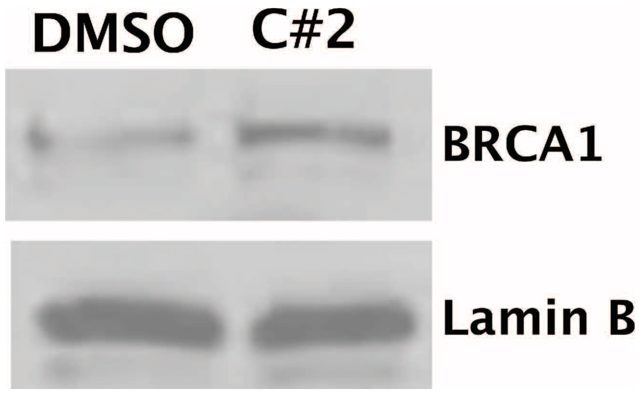

Supplemental Figure 4: C#2, direct activator of HRI induces BRCA1 expression. Cells were treated with DMSO or C#2 and expression of BRCA1 was determined by western blot analysis.
